# Temporal Lobe Epilepsy Masquerading as Panic Attacks: A Case Report

**DOI:** 10.3390/healthcare14040445

**Published:** 2026-02-10

**Authors:** Samuel Cholette-Tétrault, Philippe Leclerc, Thomas Barabé-Tremblay, Michaela Barbarosie

**Affiliations:** 1 Department of Psychiatry, Faculty of Medicine and Health Sciences, University of Sherbrooke, Sherbrooke, QC J1G 2E8, Canada; 2 Faculty of Medicine and Health Sciences, University of Sherbrooke, Sherbrooke, QC J1H 5H3, Canada; 3 Department of Psychiatry, Faculty of Medicine and Health Sciences, McGill University, Montreal, QC H3A 0G4, Canada

**Keywords:** temporal lobe epilepsy, panic attack, panic disorder, diagnosis, case report

## Abstract

**Highlights:**

**What are the main findings?**

- Temporal lobe epilepsy may mimic panic attacks, and misdiagnosis can lead to years of ineffective treatment and decreased quality of life.
- Atypical or refractory panic-like episodes could prompt neurology consultation and video-EEG evaluation, as correct diagnosis and targeted therapy markedly improve prognosis.

**What are the implications of the main findings?**

- Clinicians should maintain a high index of suspicion for neurological etiologies such as temporal lobe epilepsy when encountering panic-like symptoms, even in patients with generalized epilepsy currently under treatment.
- Early referral for neurological assessment can allow for accurate diagnosis and implementation of targeted anti-seizure therapy, resulting in improved clinical outcomes and quality of life.

**Abstract:**

**Background:** The clinical presentation of temporal lobe epilepsy (TLE) and panic disorder can sometimes overlap, particularly when the seizure symptoms include paroxysmal episodes of intense fear and autonomic symptoms. As a result, patients with TLE can be misdiagnosed with a primary psychiatric illness, which leads to inappropriate treatment, worsening of the underlying condition and decreased function and quality of life. **Clinical case:** We present the case of a 46-year-old woman, known for a 20-year history of generalized epilepsy and major depressive disorder with panic attacks that were refractory and persistent despite trials of SSRIs, benzodiazepines and cognitive behavioral therapy (CBT). While hospitalized for video-EEG monitoring in the context of worsening epilepsy, she was found to have TLE seizures presenting as what the patient had described as panic attacks, and that sometimes progressed to secondarily generalized seizures. Following a transition from a medication regimen targeting generalized epilepsy to one more appropriate for focal seizures, the patient experienced clinical improvement with a decrease in the magnitude and frequency of panic symptoms. **Conclusions:** This case, in combination with other case reports in the literature, demonstrates the need for clinical suspicion of TLE in patients presenting with atypical panic-like episodes or a refractory panic disorder, especially in cases known for epilepsy or having risk factors for seizure disorder. It also highlights the importance of comprehensive diagnostic evaluation in neuropsychiatric presentations, including EEG and brain imaging, to ensure accurate diagnosis and appropriate management.

## 1. Introduction

Temporal lobe epilepsy (TLE) is occasionally misinterpreted as panic attacks due to overlapping clinical presentations, particularly when seizures manifest as episodes of intense fear [1]. In cases of refractory panic disorder or when the clinical presentation includes known peri-ictal characteristics, such as abdominal auras, autonomic symptoms, déjà vu, or transient alterations in awareness, a neurological evaluation should be strongly considered [2,3,4,5]. Standard electroencephalography (EEG) has limited sensitivity, and long-term video EEG monitoring remains the gold standard for detecting epileptiform activity. Inability to recognize neuropsychiatric presentations triggered by epileptic disorders can lead to misdiagnosis, inappropriate psychiatric management, inadequate seizure treatment, and reduced quality of life.

## 2. Clinical Presentation

A 46-year-old woman with a 20-year history of presumed generalized epilepsy with tonic–clonic seizures and a comorbid major depressive disorder with panic attacks was admitted to an inpatient epilepsy monitoring unit (EMU) for evaluation of medically refractory epilepsy. Her first seizure occurred at age 13, when she was found by her parents in a changing room following a generalized tonic–clonic seizure with upward eye deviation and synchronous movements of all four limbs, without identifiable aura, prodrome, or trigger. Based primarily on seizure semiology, she was diagnosed with generalized epilepsy and treated long-term with valproic acid and carbamazepine, remaining on this combination for over three decades. Despite treatment, she continued to experience generalized tonic–clonic seizures approximately every three months, often occurring in the morning shortly after awakening.

In 2012 and 2013, the frequency of her seizures increased significantly to approximately 12 per month, prompting the addition of clobazam to her treatment regimen. At the time, the functional impact on the patient made it almost impossible for her to maintain employment and she reported multiple failed attempts at a return to work due to seizure frequency and unpredictability. Due to the limited benefits from the addition of clobazam, it was later tapered and discontinued after transition to adult neurology follow-up over 2019 and 2020. She experienced her longest seizure-free period in 2023, lasting several months. Over the last two to three years, the semiology of her seizures changed, with events no longer occurring predominantly in the morning and becoming less stereotyped. A notable episode in the summer of 2024 occurred during dinner following moderate alcohol intake, when she experienced a sudden fall without her typical tonic–clonic movements, with possible hypotonic posturing followed by a prolonged post-ictal state with incomprehensible speech and gesturing lasting up to four hours. She continued to deny any warning symptoms, auras, or prodromes, including déjà vu, jamais vu, epigastric rising sensations, or olfactory phenomena.

During hospitalization, she was referred for neuropsychiatric evaluation due to persistent depressive symptoms and recurrent bouts of intense anxiety. She reported unprovoked episodes of intense fear often accompanied by rising epigastric discomfort, diaphoresis, and hyperventilation, associated with irritability and social withdrawal. During these events, she would typically stop ongoing activities, become mute, flex her head forward, and cover her face with her hands. While the patient described herself as having an anxious nature and reported having had panic attacks before, the last three years were notably worse, with multiple weekly panic attacks despite trials of selective serotonin reuptake inhibitors (SSRIs), benzodiazepines, and cognitive behavioral therapy (CBT). These episodes had never been associated with convulsions and were consistently interpreted as psychiatric in nature.

At the time of admission, her antiseizure regimen consisted of valproic acid taken twice daily at doses of 500 mg and 1000 mg, propranolol 20 mg daily and carbamazepine 200 mg twice daily. A review of her medical chart revealed prior unsuccessful trials of clobazam, lacosamide, lamotrigine, and topiramate. During her EMU stay, one of her typical anxiety episodes, which she believed to be a panic attack, was captured on video-EEG, demonstrating left mid-temporal epileptiform activity coinciding with symptom onset. Brain MRI revealed no structural temporal lobe abnormalities. In light of these findings, her diagnosis was revised to focal epilepsy consistent with temporal lobe epilepsy presenting with ictal fear. Following this revision, she was transitioned from her generalized epilepsy regimen to a more targeted focal seizure management by reducing valproic acid and increasing carbamazepine to 400 mg twice daily, leading to a reduction in symptom frequency and severity with no other seizures with ictal fear manifesting during her stay on the EMU.

## 3. Discussion

### 3.1. Distinguishing Temporal Lobe Epilepsy from Panic Disorder

This case illustrates that seizure semiology in patients with established epilepsy may evolve over time to closely mimic primary psychiatric syndromes, posing a diagnostic challenge even for experienced clinicians. It further underscores that epilepsy syndromes initially presumed to be generalized, particularly when classification is based on history and semiology alone, may be misidentified, and that generalized and focal epilepsies can coexist. Accordingly, focal epilepsy, including temporal lobe epilepsy, should remain in the differential diagnosis when patients with known generalized seizures present with new or atypical neuropsychiatric symptoms. In the present case, despite appropriate neurological follow-up and recognition of neuropsychiatric complaints, these symptoms were initially interpreted as primarily psychiatric in nature, prompting psychiatric consultation before the underlying temporal lobe epilepsy was ultimately identified. This clinical course highlights the inherent difficulty of diagnosing temporal lobe epilepsy, even within specialized neurological settings.

**Figure 1:**
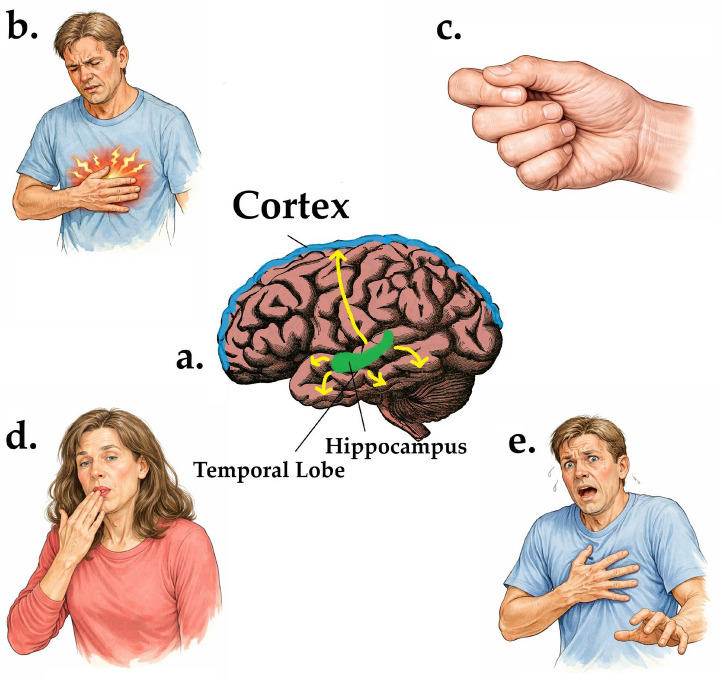
Adapted from Abarrategui et al. [7] and Chang & Lowenstein [8]. Overview of common temporal lobe epilepsy (TLE) networks and semiology. (**a**) Simplified schematic illustrating the predominant dissemination pattern of TLE (yellow arrows), with approximately 80% of seizures arising from the hippocampus before propagating to interconnected regions, including the cingulate gyrus and orbitofrontal cortex, motor and somatosensory cortices, retrosplenial cortex, and insular cortex—accounting for the broad spectrum of clinical presentations. (**b**) Illustration of a patient experiencing an epigastric aura, the most frequent aura in TLE (26–52%). (**c**) The “politician’s fist” sign is a dystonic hand posture localizing seizures to the contralateral temporal lobe. (**d**) A patient exhibiting lip smacking, a common oral automatism observed in TLE (68–91%). (**e**) A patient experiencing ictal fear, a manifestation that may be misinterpreted as a primary panic attack. GPT image version 1.5 has been used for the production and editing of the images found in (**b**–**e**).

This diagnostic challenge is critical to keep in mind, given that temporal lobe epilepsy is the most common focal epilepsy syndrome, accounting for approximately 60% of focal seizure cases, and is frequently associated with prominent psychiatric comorbidities [6]. The hallmark features of temporal lobe epilepsy include auras, loss of consciousness, and automatisms. Clinicians most often look at seizure semiology to diagnose specific subtypes. For example, lateral TLE presents with distinct features, including auditory or visual hallucinations at onset, followed by clonic movements and dystonic postures. Alternatively, mesial TLE seizures typically begin with an abdominal aura, a rising epigastric sensation often described as an uncomfortable feeling that begins in the epigastric region and ascends toward the chest or throat, and often accompanied by fear, déjà vu, jamais vu, a psychic aura characterized by a sense of unfamiliarity with a familiar situation or environment, or other neuropsychiatric symptoms, and is often followed by impaired consciousness with staring and oral automatisms such as lip smacking, chewing, or swallowing (Figure 1).

The presence of ictal fear, a discrete, often stereotyped episode of intense fear without an external trigger, is a hallmark feature of limbic seizure activity, particularly in the amygdala and hippocampus [1]. The clinical overlap between ictal fear and panic disorder poses a significant diagnostic challenge, particularly in patients without clear motor manifestations. This diagnostic challenge is highlighted by other well-documented reports of cases where temporal lobe epilepsy (TLE) was misdiagnosed as a primary psychiatric disorder [2,3]. One reported case described a pediatric patient who was initially misdiagnosed with panic disorder and was later found to have lesional temporal lobe epilepsy (TLE) secondary to a left temporal ganglioglioma [9]. Interestingly, reports of panic disorder being misdiagnosed as complex partial seizures also appear in the scientific literature, further confirming the diagnostic challenge [10].

A careful clinical history is crucial to differentiate TLE from primary panic disorder. Characteristic features suggestive of TLE over panic disorder include its prodromal rising epigastric sensation, déjà vu or jamais vu, brief altered awareness, staring spells, automatisms, and post-ictal confusion or irritability (Table 1). In contrast, primary panic disorder requires recurrent panic attacks often described as an abrupt surge of intense fear or discomfort that reaches a peak within minutes, and is accompanied by physical manifestations such as palpitations, sweating, trembling, shortness of breath, feelings of choking, chest pain, nausea or abdominal distress, lightheadedness, chills or heat sensations, paresthesias, and derealization or depersonalization. Panic attacks are usually precipitated by external stressors, are less stereotyped, lack post-episode cognitive alterations, and panic disorder is typically associated with positive response to antidepressants or CBT [11].

Moreover, findings suggestive of lesional TLE, such as hippocampal sclerosis on brain MRI and epileptiform activity on EEG, may push us towards a TLE diagnosis while keeping in mind the possibility of comorbid presentations.

### 3.2. What to Do When Suspecting Temporal Lobe Epilepsy in Psychiatric Settings

Neurological evaluation should be considered in patients with panic disorder under the following conditions:

- Refractory panic attacks that persist despite appropriate psychiatric treatment (i.e., SSRI, CBT, Benzodiazepines, etc.)
- Atypical symptoms, including epigastric aura, autonomic instability, or brief unresponsiveness
- A personal or family history of epilepsy
- Paradoxical worsening of panic symptoms with SSRIs or chronic benzodiazepine use, which can paradoxically lower seizure threshold [13]
- History of seizures or prior diagnosis of any epileptic disorder
- Known findings of brain lesions on MRI or epileptiform activity on EEG
- History of prior brain injury (e.g., hemorrhagic and ischemic stroke, traumatic brain injury, intracranial tumor, etc.)

### 3.3. Diagnostic Considerations

Although standard EEG is frequently used in epilepsy assessment, its sensitivity for detecting epileptiform activity in TLE is only 30%, particularly when seizures are infrequent [14]. Long-term video EEG monitoring in an epilepsy unit is the gold standard for diagnosis, especially in drug-resistant cases. MRI with a dedicated temporal lobe protocol can reveal structural abnormalities, including ganglioglioma [9], and mesial temporal sclerosis, found in up to 65% of patients with TLE, although a normal MRI does not exclude the diagnosis [15].

### 3.4. Consequences of Misdiagnosis

The misdiagnosis of TLE as panic disorder has serious clinical implications. Prolonged exposure to inappropriate psychiatric treatment, such as high-dose SSRIs, benzodiazepines, or psychotherapy, can delay epilepsy management, leading to increased seizure burden, chronic disability, and avoidable healthcare utilization [11]. Furthermore, untreated seizures in TLE can lead to cognitive decline, psychiatric comorbidities, and potential progression to intractable epilepsy [16].

### 3.5. Management and Treatment Considerations

Treatment strategies for temporal lobe epilepsy (TLE) should prioritize the use of appropriately targeted antiseizure medications, with an emphasis on monotherapy where feasible and combination regimens reserved for refractory cases. Among first-line options, lamotrigine is sometimes favored for its superior tolerability profile and low teratogenicity, while carbamazepine remains the most efficient option in the treatment of mesial TLE with hippocampal sclerosis, demonstrating the highest seizure freedom rate (11%), along with a high retention rate (85.9%) and rapid onset of remission [17]. Valproate is also associated with a high retention rate in MTLE-HS (85%) and remains an option in select cases of focal epilepsy [17].

In patients with drug-resistant TLE, defined by persistent seizures despite adequate trials of two or more ASMs, surgical intervention can be considered. Anterior temporal lobe resection (ATL) remains the most widely used surgical treatment, with a seizure freedom rate of 69% and superior long-term outcomes compared to minimally invasive options [18,19]. The primary risks associated with ATL include a 10.9% rate of major complications and cognitive impairments, particularly naming and verbal memory deficits when the dominant hemisphere is involved. Alternatively, selective amygdalohippocampectomy (sAHE) offers similar seizure control (66%), with a theoretical advantage in preserving lateral temporal lobe function. However, it is also associated with neuropsychological sequelae, especially in left-sided resections [19].

For patients who are not surgical candidates or who decline resective procedures, minimally invasive alternatives such as MRI-guided laser interstitial thermal therapy (MRgLITT), radiofrequency ablation (RF-TC), and stereotactic radiosurgery (SRS) may be considered, although long-term comparative data are limited [19].

Given the high prevalence of comorbid depression and anxiety in TLE, which originates from both interictal dysfunction and the psychosocial consequences of chronic seizures, a multidisciplinary approach with mental health professionals is recommended. Tailored interventions addressing mood symptoms, social functioning, and medication adherence can significantly influence overall outcomes and patient well-being.

## 4. Conclusions

Temporal lobe epilepsy should be carefully considered in patients with panic-like episodes that are resistant to first-line psychiatric treatment, that exhibit neurological features suggestive of ictal fear, or with an atypical presentation of panic disorder. Early recognition, referral to neurology and appropriate diagnostic workup, including EEG and imaging when indicated, can prevent years of misdiagnosis, reduce unnecessary psychiatric treatment, and improve patient outcomes.

## Figures and Tables

**Table 1:** Differentiating temporal lobe epilepsy from panic attacks.

	TLE	Panic Attacks
Consciousness	Impaired	Usually preserved
Duration of attack	Less than 120 s	Up to >5 min
Family history of panic attacks	uncommon	common
Antidepressant response	None or worsening	Helpful
Abnormal interictal EEG	Often present	Absent
Déjà vu & sensory auras	Common	Unusual
Anticipatory anxiety	Unusual	Common
Automatisms	Common	Unusual
Age of onset	Any age	15–30s

Adapted from Handal et al. [10] and de Lijster et al. [12].

## Data Availability

The data presented in this study are available on request from the corresponding author due to privacy restrictions.
